# Supplementary figures and images for: Detecting Corticospinal Tract Impairment in Tumor Patients With Fiber Density and Tensor-Based Metrics
Source: Front Oncol. 2021 Jan 27;10:622358. doi: 10.3389/fonc.2020.622358 (PMC7873606; doi:10.3389/fonc.2020.622358)

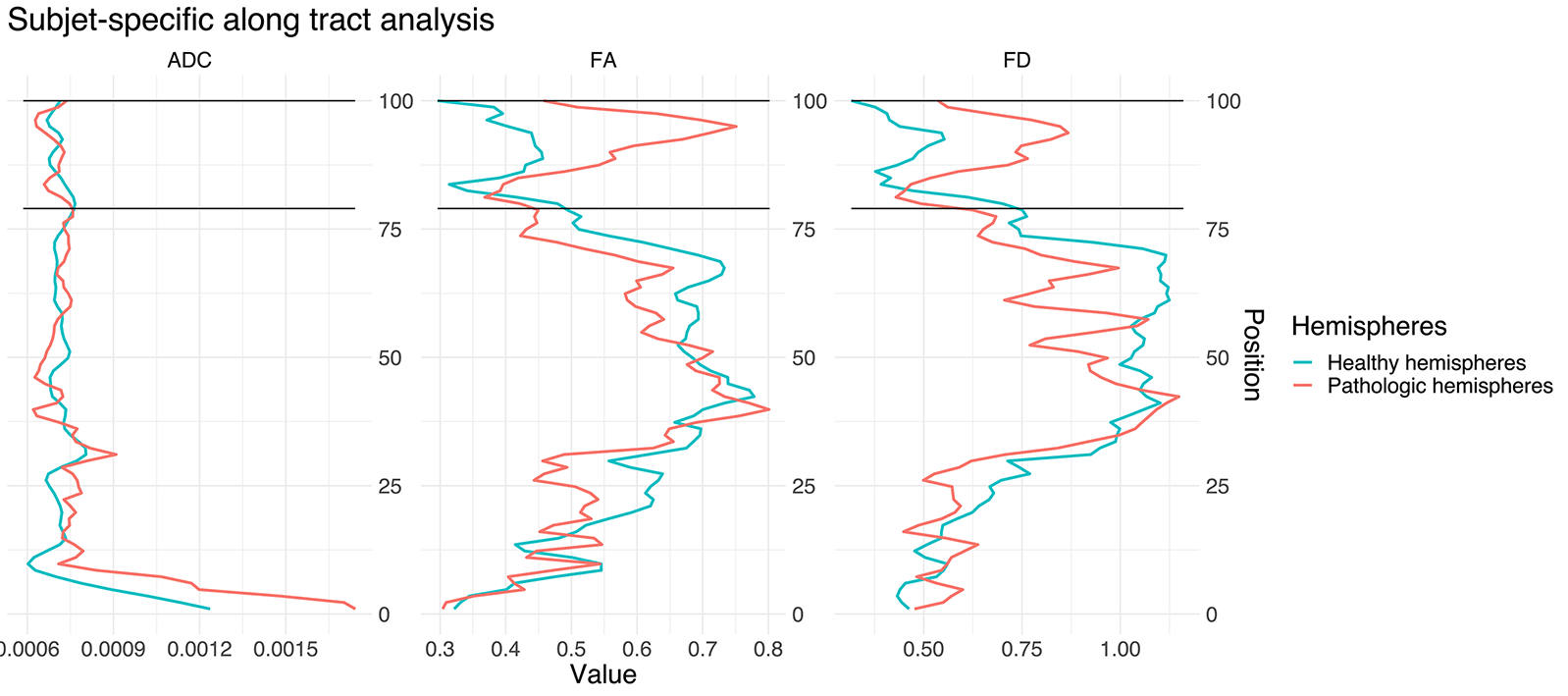

Supplement: Supplementary file 2 [file Image_1.tif]

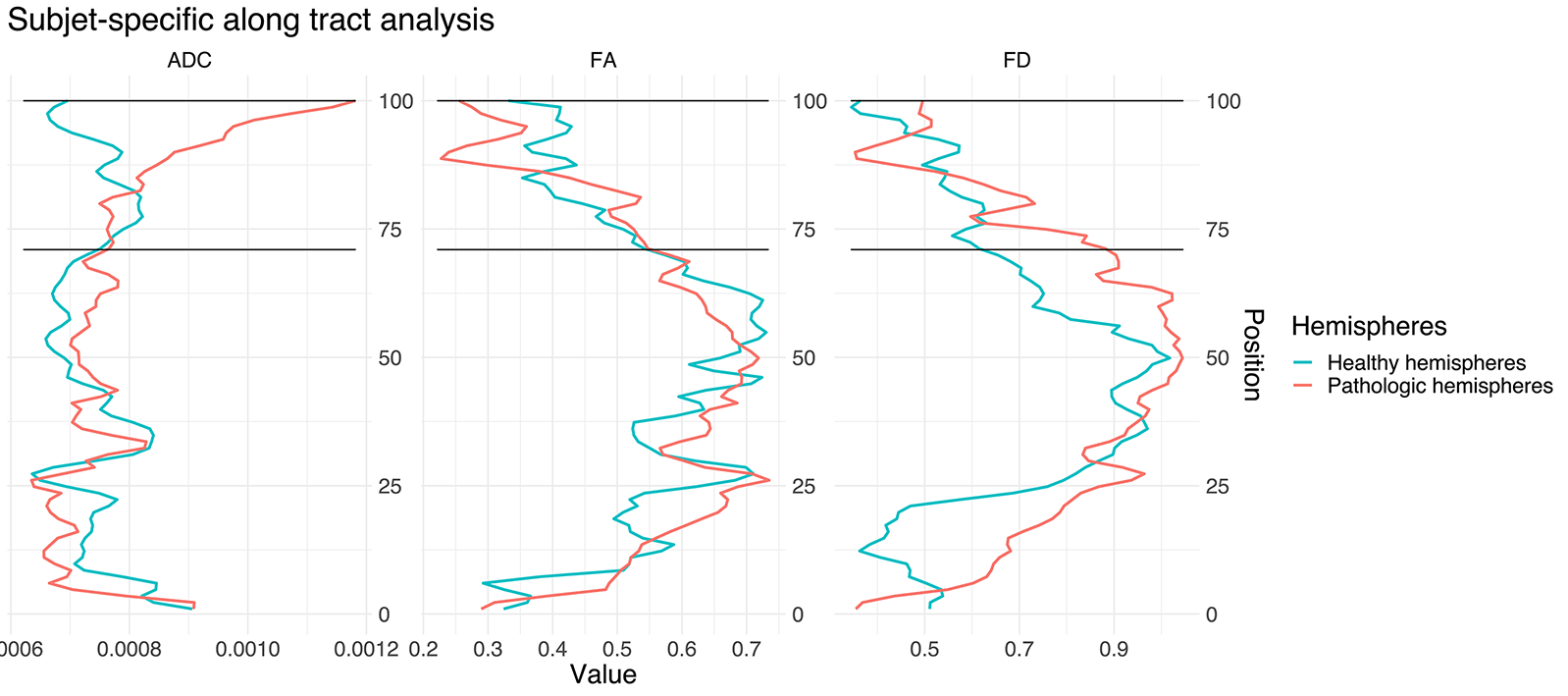

Supplement: Supplementary file 3 [file Image_2.tif]

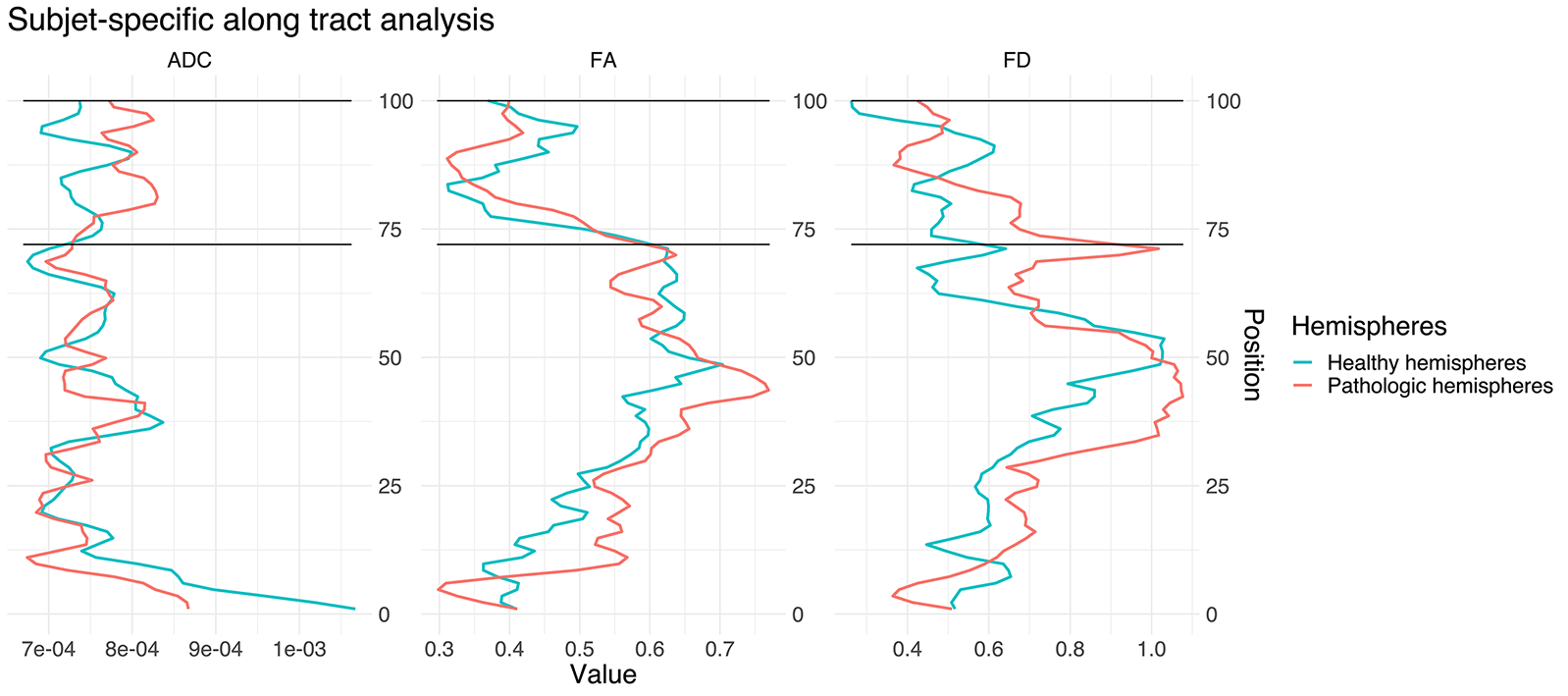

Supplement: Supplementary file 4 [file Image_3.tif]

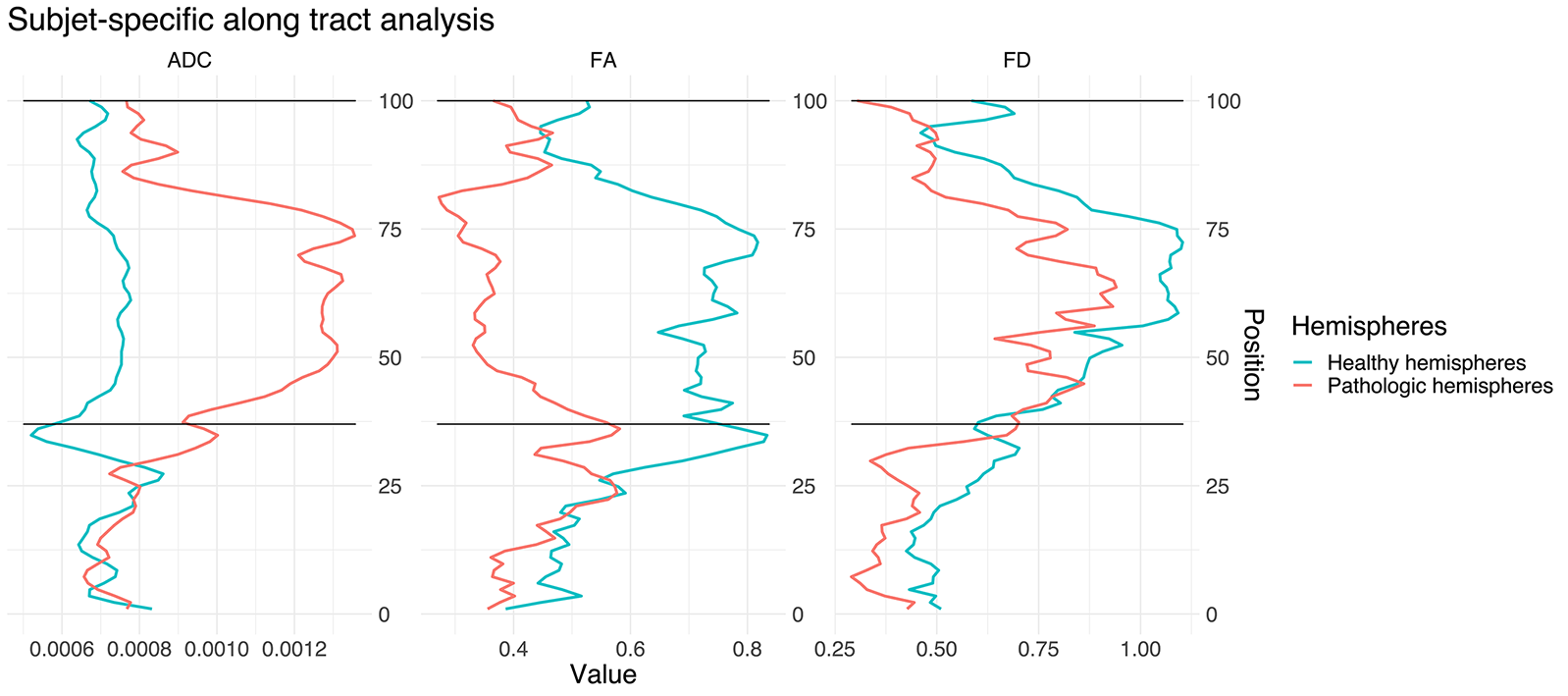

Supplement: Supplementary file 5 [file Image_4.tif]
